# Multiple Chronic Condition Combinations and Cognitive Task Performance

**DOI:** 10.1093/geroni/igaa057.1549

**Published:** 2020-12-16

**Authors:** Aaron Ogletree, Benjamin Katz

**Affiliations:** 1 American Institutes for Research, Washington, District of Columbia, United States; 2 Virginia Tech, Blacksburg, Virginia, United States

## Abstract

A growing body of literature describes important advances in the study of chronic conditions, most notably a paradigm shift from the study of individual chronic conditions to the study of multiple chronic conditions (MCCs). Despite these advances, little research has explored MCC combinations, and almost no published research has explored how MCC combinations are related to cognitive outcomes in older adult populations. Using data from the Health and Retirement Study, we categorized 17,349 older adults into one of 32 groups using self-reports of five of the most commonly diagnosed conditions. These included arthritis, diabetes, heart problems, hypertension, and respiratory problems. We utilized ANOVA to examine the associations between combinations of MCCs and performance on two cognitive tasks associated with executive function and fluid intelligence: verbal fluency and verbal analogies. Results demonstrated that older adults with a greater number of health conditions performed more poorly on both the verbal fluency (p<.0001) and analogies (p<.0001) tasks than those with fewer conditions. Some MCC combinations were associated with poorer cognitive task performance than other combinations: for example, older adults in the Heart-Hypertension-Respiratory group had an average score of 488.73 (SD=24.96) on the verbal analogies task and 14.06 (SD=7.06) on the verbal fluency task. Conversely, adults in the Arthritis-Heart-Respiratory group had average scores of 503.69 (SD=27.89) and 16.45 (SD=7.03), respectively, suggesting differential additive effects of MCCs. These findings demonstrate the complex associations of specific MCC combinations with cognitive performance and highlight the importance of better understanding the unique needs of older people with MCCs.

